# Epithelial-myoepithelial tumour of the lung: a case report referring to its molecular histogenesis

**DOI:** 10.1186/1746-1596-6-71

**Published:** 2011-07-28

**Authors:** Guillermo Muñoz, Francesc Felipo, Isabel Marquina, Celia Del Agua

**Affiliations:** 1 Department of Pathology, Hospital Universitario Miguel Servet Zaragoza, 50009, Spain

## Abstract

Tracheobronchial submucous glands can be considered the pulmonary equivalent of minor salivary glands and therefore they can develop most of the tumours originated in these. Nevertheless, in spite of the wide distribution of this kind of glands along the tracheobronchial tree, pulmonary salivary gland-like neoplasms are not very frequent. Among them, the most frequent are mucoepidermoid and adenoid cystic carcinomas. On the contrary, pulmonary neoplasms showing a mixture of epithelial and myoepithelial elements are extraordinary infrequent, with only 11 cases collected from literature.

We present the case of a 76 year-old woman with no interesting pathological history, to whom a pulmonary nodule is detected during a study of unknown origin neutropenia. An upper right lobectomy is performed.

After macro and microscopic study, the diagnosis of pulmonary epithelial-myoepithelial tumour is made. It is a low malignant potential tumour with capacity to locally recur and less frequently to metastasize. Our case has the peculiarity of not being connected neither to visceral pleura nor to bronchial tree; we have not found this characteristic in any literature reviewed case.

These tumours have been named in a lot of different ways, including adenomyoepithelioma, epithelial-myoepithelial tumour, epithelial-myoepithelial carcinoma or epithelial-myoepithelial tumour of uncertain malignant potential.

The p27/kip-1 protein plays a fundamental role in the development of these neoplasms. As we have verified in our case, its aberrant cytoplasmic location, besides its proved oncogenic function, would favour the proliferation of stem cells, which would explain both dual phenotype with presence of myoepithelial cells without connection with the bronchial tree, and TTF-1 immunostaining in epithelial cells.

## Introduction

Epithelial-myoepithelial tumours are rare neoplasms that occur more frequently in salivary glands, where they represent approximately 1% of primary tumours. In this location, they are considered as low malignant potential tumours with capacity to locally recur and less frequently to metastasize; that is why they are known as epithelial-myoepithelial carcinoma. Other sites where these neoplasms can arise are breast and skin.

We present the case of a 76 year-old woman with an asymptomatic mass in the upper lobe of her right lung, which was diagnosed as epithelial-myoepithelial tumour.

Myoepithelial cells play a fundamental role in the development of this kind of tumours. A subcellular aberrant location of p27/kip-1 inside myoepithelial cells would provoke loss of their growth-inhibition function and would contribute to tumorigenesis through lack or restriction of proliferation of myoepithelial component.

## Materials and methods

We present the case of a 76 year-old woman with no interesting pathological history, to whom a pulmonary nodule is detected during a study of unknown origin neutropenia. The patient shows good general aspect. No palpable adenopathies are detected and cardiopulmonary auscultation and all physical examinations, electrocardiogram and analysis (biochemistry, haemogram and coagulation study) do not present remarkable findings. The thoracic TC scan shows an image of a solid pulmonary nodule with polylobulated outline located in the upper right lobe (URL). Bronchoscope is normal. After posterior-lateral thoracotomy, the existence of the nodule in the URL is proved, so it is decided to perform right pneumonectomy with intraoperative biopsy. The result of it finally required an upper right lobectomy.

## Results

### Gross study

On gross examination of surgical specimen, it is seen that the nodule is located in the posterior segment of the URL and it measures 2,7 cm in its greatest dimension. The nodule is whitish, homogeneous and well delimited with regarding surrounding pulmonary parenchyma.

### Microscopic study

**Figure 1:**
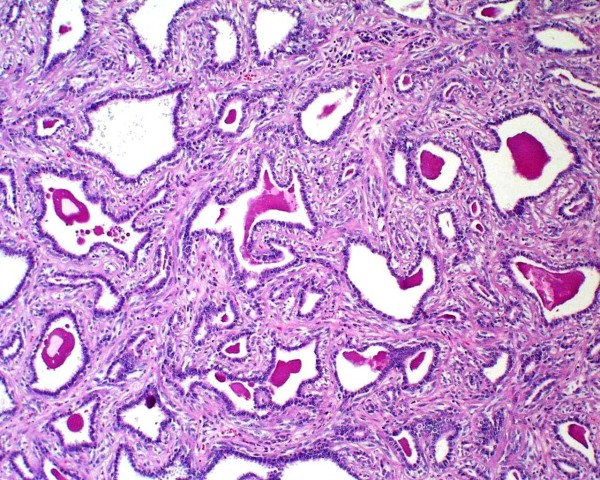
**Neoplastic cells form tubular structures mixed with little cysts and scarce solid areas**.

**Figure 2:**
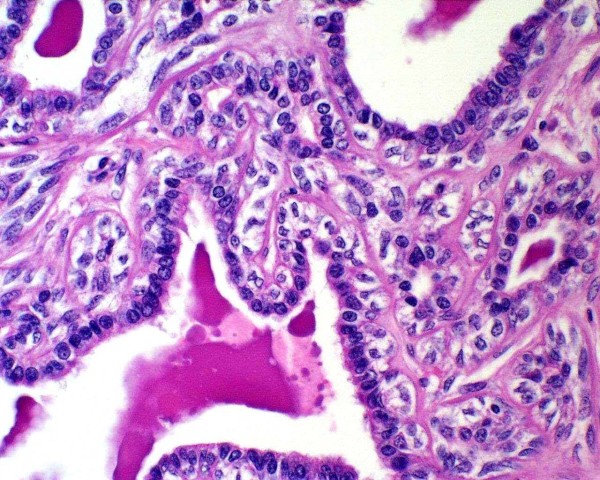
**Two cellular components are recognized, one of cuboid epithelial cells that line tubules and cysts, with eosinophilic cytoplasm and central nucleus, with patentnucleolus; the other component is from myoepithelial lineage and lies under the former; it consists of polygonal cells with abundant clear cytoplasm**.

Histologically, the tumour is well circumscribed but not encapsulated, being located into the thickness of pulmonary parenchyma, without any connection to visceral pleura or bronchial tree. Neoplastic cells are disposed forming tubular structures alternating with little cysts and scant solid areas (Figure 1). Two cellular components are recognized, one of cuboid epithelial cells that line tubules and cysts, with eosinophilic centrally located nucleus with visible nucleolus and, beneath this one, a second component from myoepithelial lineage formed by polygonal cells with abundant clear cytoplasm (Figure 2). The solid areas show a hyaline stroma with polygonal or slightly spindle cells from myoepithelial lineage. In the cystic spaces as well as in the tubular structures, a PAS-positive eosinophilic amorphous material is observed.

### Immunohistochemical study

**Figure 3:**
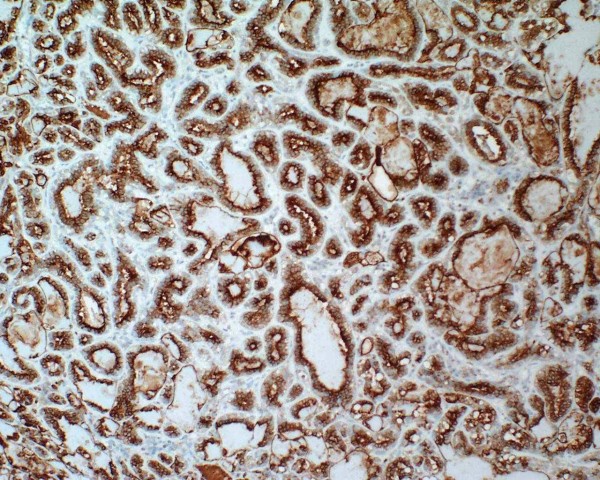
**The epithelial component is positive for keratin**.

**Figure 4:**
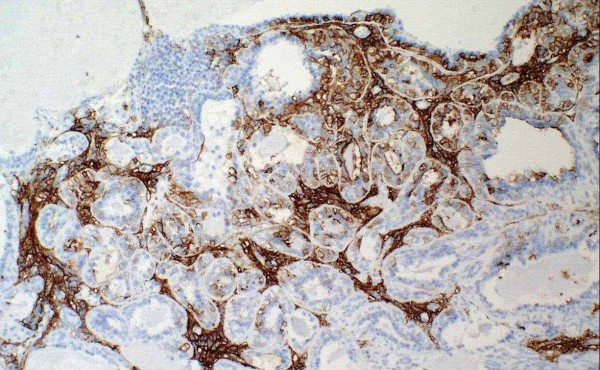
**The myoepithelial component is positive for actin**.

**Figure 5:**
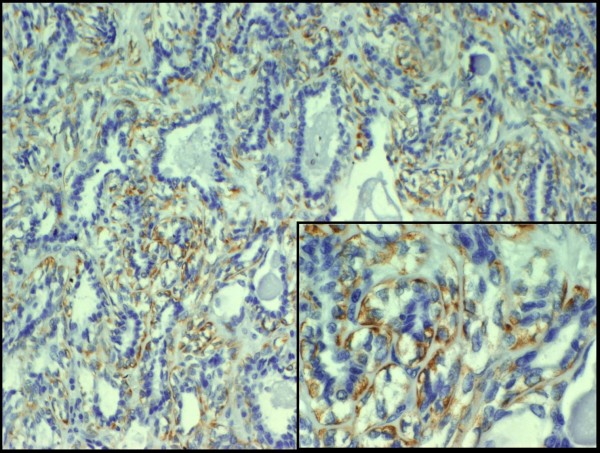
**The myoepithelial component is positive for p27/kip-1 (cytoplasmic positivity)**.

The epithelial component is positive for keratin (Figure 3), EMA and CEA, while the myoepithelial component is positive for actin (Figure 4), S-100, p63 and CD10. Epithelial cells are positive for TTF-1, unlike myoepithelial cells. It is remarkable that the latter show cytoplasmic positivity for p27/kip-1 marker (Figure 5). None of the two components show atypia or mitoses and neither necrosis nor perineural or vascular invasion are observed. With all these findings we make the diagnosis of epithelial-myoepithelial tumour of lung. Nowadays the patient is in good health and is free of disease.

## Discussion

Submucosal tracheobronchial glands can be considered the pulmonary equivalent of minor salivary glands and, therefore, they can develop most of the tumours originated from these. Nevertheless, in spite of the wide distribution of this kind of glands along tracheobronchial tree, pulmonary salivary gland-like neoplasms are not very frequent [1]. Among them, the most frequent are mucoepidermoid and adenoid cystic carcinomas. On the contrary, pulmonary neoplasms showing a mixture of epithelial and myoepithelial elements are extraordinary infrequent, with only 11 cases collected from literature. All of them came up as polypoid intrabronchial masses, although our case presents only intraparenchymal growth, with no apparent bronchial connection.

The epithelial-myoepithelial tumour of the lung appears mainly in middle-aged people, mostly females [2,1] but a case of a 7-year-old male has been reported [12]. This tumour does not seem to be related to cigarette smoking, since only 50% of the patients in the reviewed cases were smokers. The symptoms are very varied, ranging from asymptomatic cases, as the one we are presenting, to cough, haemoptysis, thoracic pain, fever [3], dyspnoea, pneumonia [4], recurrent infections and others. All patients evolved satisfactorily, without signs of recurrence nor metastases, and with no need for adjuvant chemo or radiotherapy, although recently *Nguyen et al*. have included the first description of a case of peribronchial lymph node metastases in a series of five cases [5]. One of the patients died because of a carcinoma of the floor of the mouth 36 months after the diagnose of epithelial-myoepithelial tumour of the lung was made [6].

Grossly these tumours are not encapsulated but well delimited masses, and they present an exophytic intrabronchial pattern of growth, sometimes obstructing completely the bronchial lumina. In most reviewed cases, an evident relation and connection with the bronchial tree is observed, but in others, like in our case, it presents as an intraparenchymatous mass without apparent bronchial connection [7]. It is very variable in size, ranging from 1 cm [8] to 16 cm in the case with the greatest size [6]. Its colour is whitish or white-greyish and its surface cut is white and homogeneous, not showing haemorrhage or necrosis, but exceptions [6,9].

Microscopically, the tumour was formed by tubules, cystic areas and solid areas. The proportion of these components can vary, so both components can be present in some cases [10], while in other occasions the solid component prevails [6] or, on the other hand, it does not even appear, being replaced by a hyalinized stroma [8,11]. In other cases it is the tubular component the one that abounds [4].

The tubules are lined by two cellular components, one of cuboid epithelial cells next to the lumina, and the other of myoepithelial cells beneath the first one. Both in the intercellular spaces and inside the tubules, a PAS-positive, diastase-sensitive amorphous material is observed. Necrosis is rare, but it has been described [6,9]. Exceptionally, areas of atypia and mitoses have been seen [10,9], but these mitoses are not atypical in any case. Clear cell areas [4] and squamous metaplasia [12] have also been described.

Immunohistochemically, the epithelial component is positive for keratin (Figure 3), EMA and CEA, while myoepithelial component is positive for S-100, p63 and CD10. It is remarkable the positivity of epithelial cells for TTF-1, which suggest that the tumour has a certain pneumocytic differentiation [13].

Epithelial-myoepithelial tumours of lung are considered as low malignant potential tumours with capacity to locally recur and less frequently to metastasize. It has not been described any case with vascular, lymphatic or perineural invasion, neither with distant metastasis. The disease-free survival and lack of nodal involvement correlate well with the lack of recognized histopathologic features of aggressiveness [14]. As a peculiarity, an association between these tumours and pulmonary hamartomas has been observed [14].

The differential diagnosis can be extensive and in many occasions it is going to depend on the relative predominance of myoepithelial component or on the biphasic pattern. The neoplasms that can be most frequently confused with epithelial-myoepithelial tumours of the lung are mixed tumour (pleomorphic adenoma), bronchial adenoma, adenoid cystic carcinoma, clear cell tumour of lung ("sugar tumour") and metastatic lesions, mainly those from salivary gland and kidney.

Although there is mutual agreement between the different authors about considering this neoplasia of low grade malignancy, this kind of tumour has been designated in many different ways, including adenomyoepithelioma, epithelial-myoepithelial tumour, epithelial-myoepithelial carcinoma or epithelial-myoepithelial tumour of uncertain malignant potential. Despite the fact that most cases described until now have presented no local recurrence or metastasis, and taking into account that they are generally of small size and use to lack atypia, remarkable mitotic activity, necrosis or invasive features, the metastatic potential found in some of them [5] would make the term of carcinoma epithelial-myoepithelial adequate, even when the following terms of the patients are relatively short.

*Pelosi et al*., based on a different immunostain pattern between the two neoplastic components both for ki67 and p27/kip-1, suggest that myoepithelial cells play a fundamental role in the development of this kind of tumour [3]. Protein p27/kip-1 is a cycline-dependent kinase inhibitor (CDK) that blocks cell cycle in G0 and G1. It is present in high concentration in quiescent cells and its levels slowly decrease while cells are stimulated to begin the cell cycle. Thus, p27/kip-1 inhibits and controls the progression of the cell cycle and therefore exercises a function of inhibition of tumorigenesis; in fact, it has been demonstrated that levels of p27/kip-1 are decreased in many tumours. Moreover, *Besson et al*. have recently described a dual function of this protein; as well as acting as an inhibitor of tumorigenesis, it would have oncogenic functions when it presents cytoplasmic location, acting through mediators which are little known [15]. The work also suggests that p27/kip-1 oncogenic activity leads to aberrant stem and progenitor cell expansion in the lung and retina. This study provides the first direct in vivo evidence that in addition to its role as a tumor suppressor, p27/kip-1 also functions as an oncogene. So, in epithelial-myoepithelial tumours of lung, in accordance with *Pelosi et al*. [3], an aberrant subcellular location of p27/kip-1 into the myoepithelial cell would provoke the loss of its growth-inhibition function through the lack of restriction of proliferation of myoepithelial component, which joined to the p27/kip-1's new "dark side" may serve an oncogenic function that operates in less specialized cell types to influence tumorigenesis [16].

In conclusion, we can assess that epithelial-myoepithelial tumour is a neoplasia of uncertain malignant potential, which exceptionally can arise in the lung. Our case presents the peculiarity that it has no relation with visceral pleura or bronchial tree, not having found this feature in any of the literature reviewed cases. We agree with *Pelosi et al*. that the protein p27/kip-1 plays a fundamental role in development of these tumours. As we have checked in our case, its aberrant cytoplasmic location, along with its proved oncogenic function, would favour proliferation of STEM cells, which would explain both dual phenotype with presence of myoepithelial cells without connection with bronchial tree, and TTF-1 positivity in epithelial cells.

## Consent

Despite all efforts, the patient could not be contacted to gain consent for publication of this case report. All efforts have been made to maintain anonymity.

## Competing interests

The authors declare that they have no competing interests.

## Authors' contributions

GM drafted the manuscript and participated in the final diagnosis. FF carried out the intraoperative biopsy, the gross examination and the final diagnosis. IM participated in the immunohistochemical study and in the draft of the manuscript. CDA carried out the immunohistochemical study. All authors have read and approved the final manuscript.
